#  Addressing Reemergence of Diphtheria among Adolescents through Program Integration in India

**DOI:** 10.3201/eid2703.203205

**Published:** 2021-03

**Authors:** Kiran Kumar Maramraj, M.L. Kavitha Latha, Rukma Reddy, Samir V. Sodha, Suneet Kaur, Tanzin Dikid, Sukrutha Reddy, S.K. Jain, Sujeet Kumar Singh

**Affiliations:** National Centre for Disease Control, New Delhi, India (K.K. Maramraj, S. Kaur, T. Dikid, S.K. Jain, S.K. Singh);; Ronald Ross Institute of Tropical and Communicable Diseases, Hyderabad, India (M.L.K. Latha);; State Health Department, Telangana, India (R. Reddy, S. Reddy);; Centers for Disease Control and Prevention, Atlanta, Georgia, USA (S.V. Sodha)

**Keywords:** adolescents, bacteria, children, Corynebacterium diphtheriae, diphtheria, disease outbreaks, evidence-based recommendations, immunization, vaccine-preventable diseases, zoonoses, vaccines

## Abstract

We report a diphtheria outbreak mostly among children (median 12 years; range 4–26 years) of a religious minority in urban India. Case-fatality rate (15%, 19/124) was higher among unimmunized patients (relative risk 4.1, 95% CI 1.5–11.7). We recommend mandating and integrating immunization into school health programs to prevent reemergence.

Diphtheria is a vaccine-preventable disease of the upper respiratory system caused by toxigenic strains of *Corynebacterium diphtheriae*. Global case-fatality rate (CFR) is estimated at 5%–10%; higher CFRs of up to 20% are reported in children <5 years of age (*1*). In 2016, with 78% national coverage for third-dose diphtheria-tetanus-pertussis (DTP) vaccine, India reported 48% of diphtheria cases and half of 350 deaths worldwide (*2*,*3*). In India, the 3 primary DTP doses are administered at 6, 10, and 14 weeks of age, and booster doses are given at 16–24 months and 5–6 years of age. Numerous states across India have reported diphtheria outbreaks, including Assam in 2010, Karnataka in 2011, and Andhra Pradesh in 2014 (*4*). In December 2017, the Integrated Disease Surveillance Program of Telangana state reported a rise in diphtheria cases. We investigated to describe the epidemiology of the outbreak, identify risk factors, assess trends in immunization coverage, and provide evidence-based recommendations. 

## The Study 

**Figure 1:**
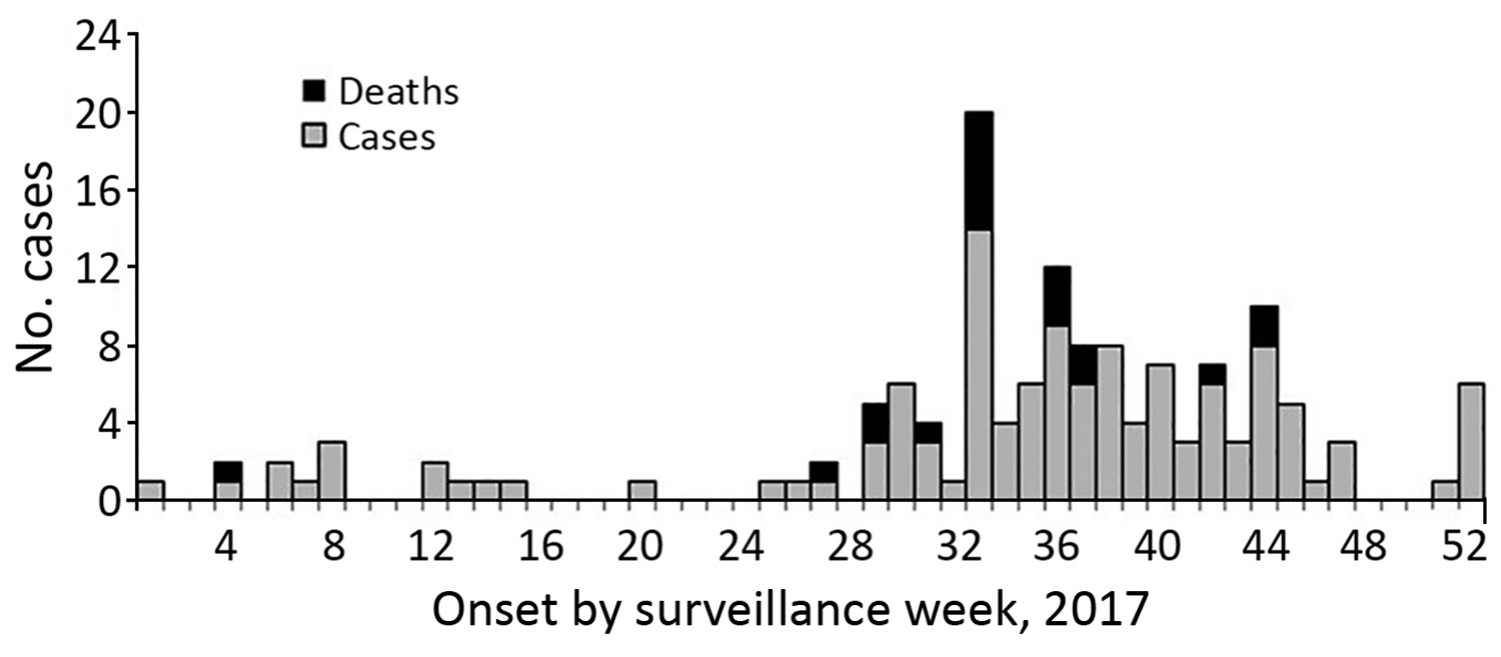
Epidemic curve by patient date of illness onset for 124 confirmed diphtheria cases, including 19 deaths, Telangana, India, 2017.

For this study we defined a diphtheria case as an upper respiratory tract illness with an adherent pseudomembrane in the nasal cavity, pharynx, or larynx and *C. diphtheriae* isolated from a clinical specimen from a Telangana resident during January 1–December 31, 2017. Clinical specimens were cultured initially on blood tellurite medium followed by selective culture on cystinase medium. We identified 124 laboratory-confirmed diphtheria cases, for an annual incidence of 3.5 cases/1 million residents; the 19 deaths represented a CFR of 15%. This incidence was more than the mean incidence +2 SD of 2.9 cases/1 million residents during 2014–2016, which confirmed the 2017 cases as an outbreak. Age range for case-patients was 4–26 years (median 12 years). Adolescents 10–14 years of age had the highest annual incidence rate, 15/1 million residents. CFR decreased by age from 24% among children 4–9 years of age to no deaths in persons 20–29 years (odds ratio [OR] 1.9, 95% CI 1.1–3.4; p = 0.03). Only 11% (14/124) of laboratory-confirmed samples had an Elek test for toxigenic strain; 12 (86%) of those 14 samples were positive. Female patients accounted for 50% of cases but 63% of deaths. Children identified as Muslim, a religious minority in Telangana, accounted for 60% of cases, but 74% of deaths. Most cases (81%) and deaths (89%) occurred in the last half of 2017 (Figure 1). Urban Hyderabad makes up only 11.2% of the population of Telangana (https://www.telangana.gov.in/PDFDocuments/Statistical-Year-Book-2017.pdf) but accounted for 53% of diphtheria cases and 47% of deaths in the state; annual incidence in Hyderabad was 19 cases/1 million population, the highest among all geographic areas of Telangana. This investigation was a public health response to an outbreak. Requisite approvals were obtained from national and state health authorities. 

We conducted a retrospective cohort study to assess factors associated with death among case-patients. We defined the cohort as all patients with laboratory-confirmed diphtheria in Telangana during January 1–December 31, 2017. Among 124 case-patients identified, 25 (20%) were not located or declined to provide immunization information; 99 (80%) patients participated in the cohort study. Among the 99 patients, immunization coverage for DTP3 was 53% and for DTP second booster was 36%, based on vaccination card or parental recall when the card was not available (Table 1). Case-patients without all 3 doses of the primary immunization series were more likely than those having had the full DPT3 to die from diphtheria (relative risk [RR] 4.1; 95% CI 1.5–11.7) with 60% attributable risk. Symptoms significantly associated with death were hoarseness (100%), dyspnea (100%), bull neck appearance (89%), and stridor (42%) (p <0.001 for all). Delayed hospital admission (i.e., >72 hours elapsed after sore throat onset) was also significantly associated with death (RR 2.8, 95% CI 1.2–6.8) (Table 2). 

**Table 1:** Immunization status of 99 study patients with confirmed diphtheria cases, Telangana, India, 2017*

Diphtheria vaccine	Based on card information only, no. (%)	Based on card information or parental recall, no. (%)
Pentavalent 1 or DTP1	23 (23)	55 (56)
Pentavalent 2 or DTP2	23 (23)	53 (54)
Pentavalent 3 or DTP3	23 (23)	52 (53)
DTP booster 1	21 (21)	44 (44)
DTP booster 2†	17 (18)	35 (36)

*DTP, diphtheria-tetanus-pertussis vaccine. †Denominator for DTP booster 2 = 96; three 4-year-olds were excluded because they were not eligible for DTP booster 2 based on age.

**Table 2:** Risk factors for mortality for 99 study patients with confirmed diphtheria cases, Telangana, India, 2017

Risk factor	Died, no. (%), n = 19	Survived, no. (%), n = 80	CFR, %		RR (95% CI)	p value
			Among exposed	Among nonexposed		
Not fully immunized with DTP3	15 (79)	32 (40)	32	8	**4.1 (1.5–11.7)**	0.002
Delayed hospital admission	13 (68)	30 (38)	30	11	**2.8 (1.2–6.8)**	0.01
<10 y of age	12 (63)	32 (37)	27	13	2.1 (0.9–4.9)	0.06
Muslim	14 (74)	48 (60)	23	14	1.7 (0.7–4.3)	0.27
Female	12 (63)	40 (50)	23	15	1.5 (0.7–3.6)	0.30
Rural residence	10 (53)	38 (47)	21	18	1.2 (0.5–2.6)	0.68

*Bold indicates statistical significance. CFR, case-fatality rate; DTP, diphtheria-tetanus-pertussis vaccine; RR, relative risk.

**Figure 2:**
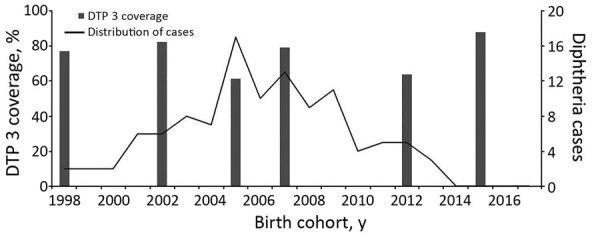
Distribution of DTP3 immunization coverage during 1998–2016 and hypothetical distribution of 2017 cases according to birth cohorts in Telangana, India. Data sources for DTP-3 coverage are National Family Health Surveys (NFHS; *5*) and District Level Household & Facility Surveys (DLHS; *6*), conducted by the government of India. Data source for coverage in 1998 was NFHS-2, in 2005 was NFHS-3, and in 2015 was NFHS-4. Data source for coverage in 2002 was DLHS-2, in 2007 was DLHS-3, and in 2012 was DLHS-4. DTP3, diphtheria-tetanus-pertussis.

We reviewed DTP immunization coverage trends in Telangana during 1998–2016 by assessing National Family Health surveys conducted in 1998–1999, 2005–2006, and 2015–2016 (*5*) and District-Level Household and Facility Surveys conducted in 1998–1999, 2002–2004, 2007–2008, and 2012–2013 (*6*). DTP3 coverage showed a dip in 2005 (61% in 2005 vs. 75% mean during 1998–2016). The diphtheria cases reported in 2017 were hypothetically distributed according to their birth cohorts over the period 1998–2016, to compare with the immunization coverage of that year. Around half of these cases (48%, 60/124) occurred during 2005–2009, after a dip in immunization in 2005 (Figure 2).

We assessed the available records during 2014–2017 from 12 healthcare facilities in urban Hyderabad. None had periods when vaccines were out of stock, all had cold chain temperature logs maintained within the appropriate range, and all conducted >80% of the immunization sessions across all quarters; administrative immunization reported >90% DTP3 coverage. Interviews of health facility staff revealed that all 12 facilities had an immunization-tracking system in place for children <2 years of age. However, for children ≥2 years of age, there was no tracking mechanism, and they were not included in the routine coverage surveys and administrative coverage reports. Mission Indradhanush*,* a nationwide immunization drive by the government of India, has made major gains in improving immunization coverage; however, it did not target children ≥2 years of age (*7*).

Our study is limited because probable cases of diphtheria not confirmed by laboratory testing and asymptomatic cases were excluded, so the outbreak was likely underestimated. In addition, we did not conduct population immunization coverage surveys in the affected community and relied on published government estimates instead. 

## Conclusions

The age shift of diphtheria cases is of global concern. Case-based surveillance studies in India have suggested that areas with greater immunization coverage have experienced an age-shift with a higher incidence among older children (*8*,*9*). In this diphtheria outbreak, cases were primarily among adolescents and school-age children; no cases were reported in children <4 years of age, probably because of high (>90%) vaccine coverage in birth cohorts since 2014. Gaps in booster-dose coverage probably resulted in waning immunity provided by the primary series (*10*,*11*). This outbreak had a much higher CFR (15%) compared with the national CFR of 3% for diphtheria in 2017 (*12*). CFR was higher among underimmunized children and those with delayed hospital admission, similar to previously reported outbreaks (*13*–*15*). Hyderabad reported incidence 5 times higher than the average in the state. The Muslim community makes up only for 12% of Telangana’s population but accounted for 60% of cases and 74% of deaths due to diphtheria reported in the state. 

To address the factors leading to this outbreak and to prevent diphtheria outbreaks in the future, we recommended 2 main strategies. First, we recommend adding 2 adolescent booster doses at 10 and 16 years of age to the routine immunization schedule, which would address possible waning of immunity from the primary series. To help accomplish this, we recommend integrating the immunization program with school health programs. Schools annually identify and track eligible schoolchildren for administration of age-appropriate vaccine doses. The government could mandate that schools require a second DTP booster before students enter primary school (ages 5–6 years) and a tetanus-diphtheria booster as they leave primary school (ages 10–11 years) and secondary school (ages 15–16 years). Second, we recommend implementing focused immunization services in urban Muslim communities by engaging religious leaders and community stakeholders. Addressing gaps in routine delivery of immunization service in marginalized and underserved populations is essential for averting future vaccine-preventable disease outbreaks. 
